# Integrating narrative mentorship into simulation-based medical education to support professional identity formation

**DOI:** 10.5339/qmj.2026.49

**Published:** 2026-08-11

**Authors:** Ali Msheik, Hassan Al Thani, Ruben Peralta

**Affiliations:** 1 Neurosurgery, Neuroscience Institute, Hamad Medical Corporation, Doha, Qatar; 2 Department of Surgery, Trauma Surgery, Hamad Medical Corporation (HMC), Doha, Qatar; 3 Department of Surgery, School of Medicine, Universidad Nacional Pedro Henríquez Ureña, Santo Domingo, Dominican Republic

## 1. INTRODUCTION

**Figure 1.**
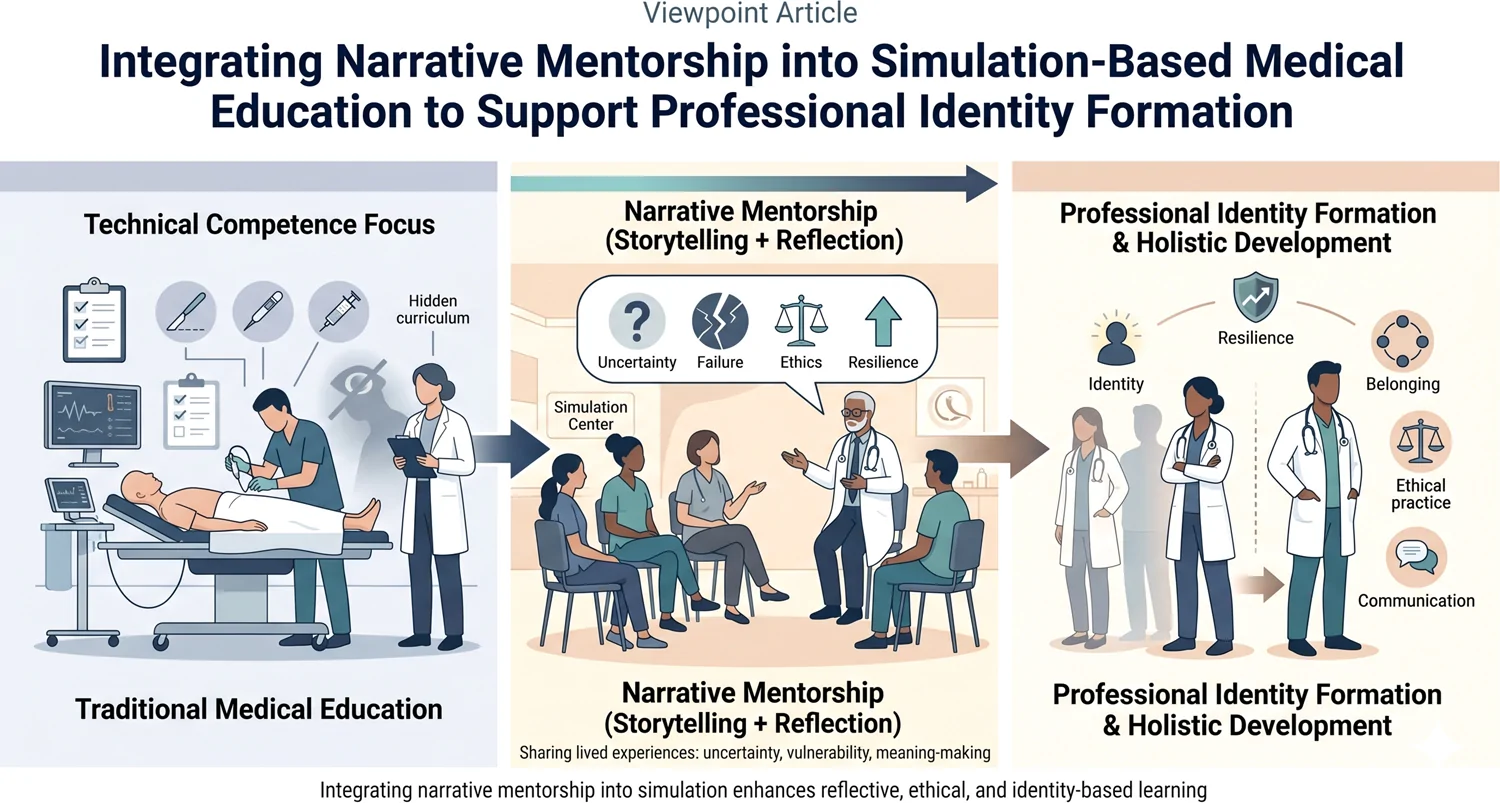
Graphical summary of the study rationale, methodology, key findings, and conclusions.

Medical education traditionally prioritizes technical competence. Research increasingly highlights professional identity formation (PIF) and the hidden curriculum as fundamental influences on how learners understand their role in healthcare.^1,2^ These dimensions shape learner behavior and wellbeing, but remain inconsistently addressed in training environments. A candid narrative shared by a senior clinician illustrated the influence of narrative mentorship, defined here as a reflective mentoring approach in which experienced clinicians intentionally share personal clinical experiences, including uncertainty, failure, ethical tension, and meaning-making, to support learners’ identity development. Unlike traditional mentorship, which often emphasizes career guidance, performance feedback, and skills acquisition, narrative mentorship centers on storytelling as a pedagogical tool to normalize vulnerability, model professional values, and facilitate reflective dialogue. This viewpoint examines the educational implications of clinician narratives and the value of integrating narrative mentorship into simulation-based programs. Narrative mentorship remains underused in contemporary medical education, despite strong potential to address gaps in professional identity formation and learner wellbeing. An overview of the study rationale, methodology, and principal findings is presented in Figure 1.

## 2. NARRATIVE MENTORSHIP AS A CATALYST FOR IDENTITY FORMATION

More than 700 students attended a national educational session at ITQAN (Hamad Medical Corporation’s national simulation and clinical skills center) during a national medical education symposium,^3^ reflecting strong interest in mentorship and professional development. The keynote speaker shifted from conventional institutional messaging to a candid account of early-career challenges, uncertainty, and resilience. He highlighted the role of mentorship and early formative experiences requiring resilience. Learners’ questions centered on belonging, coping, and identity formation, reflecting educational needs that are not consistently or explicitly addressed within many competence-based curricula internationally.^2,4^ Narrative mentorship allows clinicians to articulate uncertainty, vulnerability, and meaning, dimensions often peripheral to formal competency frameworks but recognized as influential in professional identity formation.

## 3. EDUCATIONAL IMPLICATIONS OF CLINICIAN NARRATIVES

The surgeon’s account demonstrated how narrative mentorship can deepen reflective, attitudinal, and professional learning rather than directly teaching procedural knowledge or technical skills.^5^ Openness challenged common assumptions in the hidden curriculum that successful clinicians are always confident and composed.^4,6^ This honesty supported professional identity formation by validating learners’ fears. While acquisition of surgical or procedural skills requires deliberate practice and technical supervision, narrative mentorship may complement this process by strengthening resilience, ethical reasoning, communication, and tolerance of uncertainty, attributes that influence how technical competence is enacted in clinical practice. Such moments can reinforce purpose, belonging, and meaning for learners at all stages of training.

## 4. IMPLICATIONS FOR EDUCATORS

Educators should integrate narrative sessions into simulation programs to complement technical learning and promote reflective practice. Faculty development in narrative competence can enhance the intentional use of storytelling, while structured debriefing allows learners to explore uncertainty, emotional responses, and professional values. Simulation centres offer psychologically safe environments for such intergenerational dialogue.

## ACKNOWLEDGEMENTS

We would like to acknowledge the following individuals and teams for their volunteerism: Wedad Qassim S.M. Al-Muraysi, Ana Nafra, Gursharnpreet Kaur, and The Health Professions Awareness and Volunteering Program (HPAP), ITQAN - Hamad Medical Corporation (HMC).

## AUTHOR CONTRIBUTIONS

AM, RP: Conceptualization; AM, RP: Methodology; AM: Investigation; AM, HAT: Data curation; AM, HAT: Formal analysis; AM, HAT, RP: Writing—original draft; All authors: Writing—review and editing; HAT, RP: Supervision. All authors reviewed and approved the final version of the manuscript.

## CONFLICT OF INTERESTS

The author(s) declare that there is no conflict of interest.

## DISCLOSURE OF AI USE

Generative artificial intelligence tools, including OpenAI ChatGPT/Codex, were used solely for language refinement, formatting, and editorial assistance. These tools were not used to generate or modify study data, perform the statistical analyses, or determine the scientific conclusions. All AI-assisted content was critically reviewed and verified by the authors, who take full responsibility for the accuracy and integrity of the manuscript.

## DATA AVAILABILITY

All data supporting the findings of this study are available within the published article and its supplementary materials. No additional unpublished datasets were generated.

## FUNDING

The author(s) received no financial support for the research, authorship, and/or publication of this article.

## ETHICS APPROVAL

Ethics committee approval and informed consent were not required because this study was based exclusively on previously published literature and involved no new human participants, identifiable patient information, or animal experimentation.
